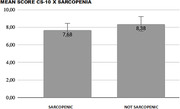# Do patients in early‐stage cognitive impairment have an increased risk of developing sarcopenia? A cross‐sectional study

**DOI:** 10.1002/alz.089351

**Published:** 2025-01-09

**Authors:** Pedro de Castro Lopes, Amanda Aparecida Oliveira Leopoldino, João Carlos Barbosa Machado, Maira Tonidandel Barbosa, Luana Rodrigues Garcia, Bianca Pessoa Aguiar, Júlia Caroline Barbosa de Souza, João Pedro Neres Antunes Ferreira

**Affiliations:** ^1^ Rede Mater Dei de Saúde, Belo Horizonte, Minas Gerais Brazil; ^2^ Faculdade Ciências Médicas de Minas Gerais, Belo Horizonte, Minas Gerais Brazil

## Abstract

**Background:**

Sarcopenia is a prevalent condition defined by a loss of muscle mass associated with reduction in muscle power and physical performance. It is associated with several adverse outcomes such as risk of falls and fractures, hospitalization and higher mortality. It can be triggered by several clinical conditions and dementia is one of them. Many studies point the relationship between cognition and sarcopenia, especially in advanced cases of dementia. However, more studies are necessary to understand whether patients with mild cognitive impairment have also increased risk for developing sarcopenia.

**Method:**

This is a cross‐sectional study in which 87 community‐dwelling elderly people were included and evaluated in relation to the presence of cognitive impairment and sarcopenia. Patients with severe cognitive impairment, assessed by a score less than 6 in the CS‐10 (Point Cognitive Screening) were excluded. Individuals with a score between 6 and 7 on the CS‐10 were considered to have mild or moderate cognitive impairment and participants with a score greater than 7 on this test were considered to have no cognitive impairment. In order to evaluate the presence of sarcopenia it was applied the SARC‐F CC questionnaire. Those with a score greater than or equal to 11 were considered sarcopenic and a score between 0 and 10 without sarcopenia. With the aim of estimate the association between the characteristics, Pearson’s chi‐square test was calculated. If the p‐value was lower than the significance level of 0.05, it is possible to conclude the correlation between the development of sarcopenia and a worse score in CS‐10.

**Result:**

In the sample studied, 29.1% were classified with an increased risk for sarcopenia. The mean CS‐10 score was 8.2 with a standard deviation of 1.7 points. Patients with higher SARC‐F CC scores had a lower mean score on the CS‐10, but these results did not show a statistically significant difference (p‐value 0.232).

**Conclusion:**

Despite of a tendency for patients with mild cognitive impairment to have a higher risk for developing sarcopenia, it is not possible to affirm this relationship from this study, since no statistically significant association was identified.